# An Improved Diagnostic of the *Mycobacterium tuberculosis* Drug Resistance Status by Applying a Decision Tree to Probabilities Assigned by the CatBoost Multiclassifier of Matrix Metalloproteinases Biomarkers

**DOI:** 10.3390/diagnostics12112847

**Published:** 2022-11-17

**Authors:** Anastasia I. Lavrova, Eugene B. Postnikov

**Affiliations:** 1 Saint-Petersburg State Research Institute of Phthisiopulmonology, 194064 Saint Petersburg, Russia; 2 Sophya Kovalevskaya North-West Mathematical Research Center, Immanuel Kant Baltic Federal University, Nevskogo st. 14, 236041 Kaliningrad, Russia; 3 Theoretical Physics Department, Kursk State University, Radishcheva st. 33, 305000 Kursk, Russia

**Keywords:** tuberculosis, machine learning-based diagnostics, matrix proteinases, CatBoost

## Abstract

In this work, we discuss an opportunity to use a set of the matrix metalloproteinases MMP-1, MMP-8, and MMP-9 and the tissue inhibitor TIMP, the concentrations of which can be easily obtained via a blood test from patients suffering from tuberculosis, as the biomarker for a fast diagnosis of the drug resistance status of *Mycobacterium tuberculosis*. The diagnostic approach is based on machine learning with the CatBoost system, which has been supplied with additional postprocessing. The latter refers not only to the simple probabilities of ML-predicted outcomes but also to the decision tree-like procedure, which takes into account the presence of strict zeros in the primary set of probabilities. It is demonstrated that this procedure significantly elevates the accuracy of distinguishing between sensitive, multi-, and extremely drug-resistant strains.

## 1. Introduction

Tuberculosis (TB) is a recent infectious disease that still places a heavy burden on worldwide public health. as is reported in the current issue of the *Global Tuberculosis Report 2021* [1] by the World Health Organization, annually, the number of TB diagnoses reaches about 10 million people and about 1.3 million deaths. The situation is complicated by the active development of drug resistance of *Mycobacterium tuberculosis* (MtB), which causes this illness, to conventional (first-line) drugs and even to all known kinds of medications, i.e., multidrug resistance (MDR) and extremal drug resistance (XDR) [2,3].

At the same time, it is worth noting that revealing the drug resistance status via methods using growth and drug resistance testing of pathogen culture is a time-consuming procedure as, in most cases, urgent therapy is needed (depending on the resistance of the strain of *M. tuberculosis*). This supports a demand for alternative methods [4,5,6]. Among such methods, special attention is paid to the usage of specific biomarkers [7,8,9,10].

**Figure 1:**
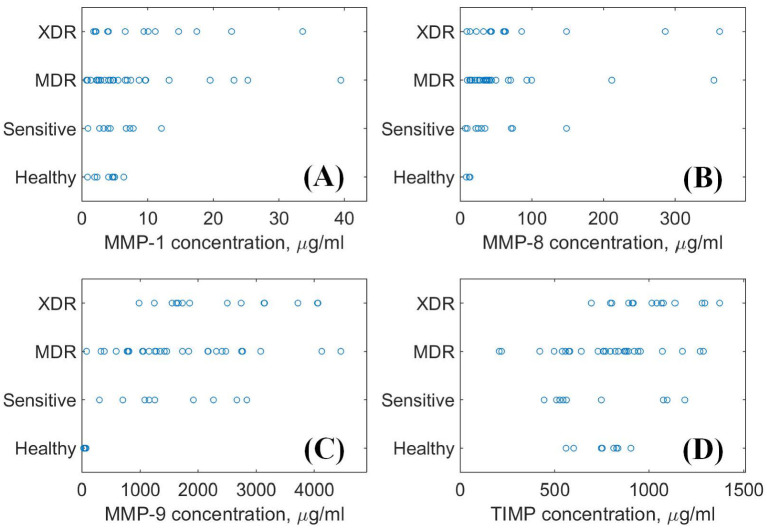
The distribution of the biomarker ((**A**): MMP-1, (**B**): MMP-8, (**C**): MMP-9, (**D**): TIMP) concentrations sorted with respect to the drug resistance status. The data are taken from the freely available source [16] (published under the Creative Commons Attribution (CC BY) license).

The number of biomarkers specific to the development of TB-based tissue destruction includes matrix metalloproteinases (MMPs) and their specific tissue inhibitors (TIMPs) [11,12,13,14]. It should be noted that MtB plays the role of a “wire-puller” in the operating activity of three principal MMPs, namely MMP-1, MMP-8, and MMP-9, as well as TIMP-1 (further referenced simply as TIMP). as a consequence, the concentration of these biomolecules can serve as potential biomarkers [13,15]. Recently, a relatively large dataset of MMPs and TIMP concentrations obtained from patients with the confirmed various drug resistance status of MtB has been published [16]. This opens up prospects for their evaluation as potential indicators to distinguish between sensitive, MDR, and XDR forms, as well as between healthy and infected persons. However, as seen in Figure 1, these concentrations are widely scattered, which makes it impossible to introduce simple analytic correlations. To overcome this difficulty, machine learning (ML), which may reveal complex hidden dependencies, is a promising candidate for processing these data.

Among different ML approaches, we have chosen the CatBoost algorithm [17] for our stated goal. Classification and multiclassification are natural features of this approach, which is based on gradient boosting on decision trees and has been made publicly available, thereby gaining increasing popularity in different research applications [18], including those related to biomarker-based decisions [19,20,21,22].

In our preliminary work [23], we demonstrated that the simplified binary classification with CatBoost is relatively reasonably (with 75% accuracy) able to distinguish between sensitive and drug-resistant strains. Thus, this work aims at a deeper investigation of this problem from the point of view of a detailed multiclassification of the types of drug resistance and how they differ from the MMP/TIMP status of patients and healthy persons.

In addition, note that the program implementation of CatBoost provides a high level of detail in the output results on which a decision is based. It allows for operation with the concept of interpretable machine learning [24,25], not only supporting the assurance of ML-based clinical decisions and elevating the accuracy of predictions but also helping to reveal the biochemical mechanisms of the respective processes.

## 2. Materials and Methods

### 2.1. Dataset

The data used for processing in this work are taken from the previously published, freely available experimental dataset [16]. It contains clinical data obtained from 234 patients with pulmonary tuberculosis (TB) induced by MtB with different confirmed resistance statuses treated at the Saint Petersburg State Research Institute of Phthisiopulmonology (SRIP) during the time period of 2009–2017 as well as from a control group of healthy persons. a detailed description of this dataset is provided in the cited work; here, we used the numerical values of the concentration of MMPs and TIMP for their analysis by means of machine learning.

Note that the complete dataset reported in work [16] contains multiple lacunas in its data, i.e., missing values of concentrations of the required biomarkers. Although the CatBoost algorithm, in principle, allows for the use of datasets with missing values of input parameters, in the present work, we use results from the whole dataset, which include the full sets of data on all four metalloproteinases, to avoid artificial uncertainty related to the processing of the missing data. The resulting reduced dataset includes data for the control group of 8 healthy persons, 9 persons infected with the MtB form sensitive to conventional drugs, 27 with multiple drug-resistant forms, and 14 extremely drug-resistant MtBs. as biomarkers, three types of matrix metalloproteinases (MMP-1, MMP-8, MMP-9) and the related tissue inhibitor TIMP were chosen from the variety of available parameters. Figure 1 demonstrates the distribution of their concentrations grouped according to the drug resistance status of the TB-suffering patients and the control group of healthy persons. One can see wide ranges of concentration dispersion for almost every group and biomarker.

Therefore, in this case, using a simple regression is unsuitable. The use of machine learning methods is required, which, in principle, can reveal some possible hidden patterns and, as a result, provide a way to classify data as corresponding to different MtB drug resistance statuses.

### 2.2. Machine Learning Algorithm and Its Implementation

We used the free software CatBoost version 0.26 (https://catboost.ai/, accessed on 16 November 2022) with the command line compiled version as the machine learning-based multiclassification system. The input tables for the training of MMP concentrations were passed to the function ’fit’ for computation as follows: catboost-0.26.exe fit –learn-set trainDataNum.txt –column-description TabDescr.txt –loss-function

MultiClass. Here, the abovementioned text file containing a tabular-separated table, trainDataNum.txt contains five columns of numerical data. The first column reports the resistance status encoded as numbers from 0 (healthy) to 3 (1, 2, 3 mean SENS, MDR, XDR, respectively), and the second through fifth columns contain the concentrations of MMP and TIMP. a description of this structure is given in the file TabDescr.txt, which has the content:

0 Label;

1 Num TIMP;

2 Num MMP-1;

3 Num MMP-8;

4 Num MMP-9.

The switch option MultiClass for the loss functions causes the application of the CatBoost multiclassification algorithm with default settings, which are known to be optimised for this class of machine learning problems [26]. This procedure generates the binary file model.bin, which contains the trained model.

For the predictive multiclassification, the command-line string catboost-0.26.exe calc -m model.bin –input-path testDataNum.txt –prediction-type Class, Probability -o Out.tsv was carried out. This refers to the model and the input data contained in the text file testDataNum.txt, which have the same tabular structure as the file trainDataNum.txt but without the first columns, i.e., without the known classes, which should be generated now by the trained model. To denote this, the switch –prediction-type is supplied with the Class option. The second option, Probability reports the explicit values of the probabilities assigned by the CatBoost model to each class, which will be used to improve the classification. The output data are saved to the tabular-separated table in the text file Out.tsv.

Since the amount of available clinical data is not very large, we used the complete permutation cross-validation proposed and discussed in work [27] to evaluate the accuracy of the prediction procedure. The essence of this approach is the following: One set of data is excluded sequentially from the full dataset, and all the rest are used to train the model. After this, the trained model is applied to the prediction of the drug resistance status of this excluded input dataset. Practically, this means that the file trainDataNum.txt contains 57 of the 58 total strings of the full table, and one string is transferred to the file testDataNum.txt, where it is alone. The procedure repeats while the complete permutation of all data is reached. This approach avoids the uncertainty related to the subdivision of small datasets into small-size training and testing sets and obtains the number of tests equal to the size of the available data without overlapping the training and test datasets.

## 3. Results

### 3.1. Failure of the Default (Naïve) Multiclassification

**Figure 2:**
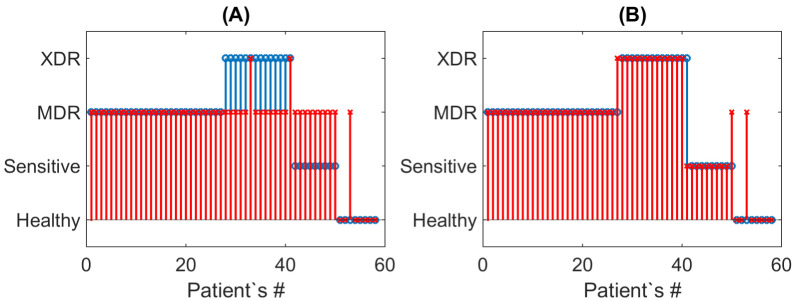
A comparison of the drug resistance statuses for the naïve (**A**) and improved (**B**) multiclassification approaches. Blue stems with circles and red stems with crosses mark the actual and predicted outcomes.

**Figure 3:**
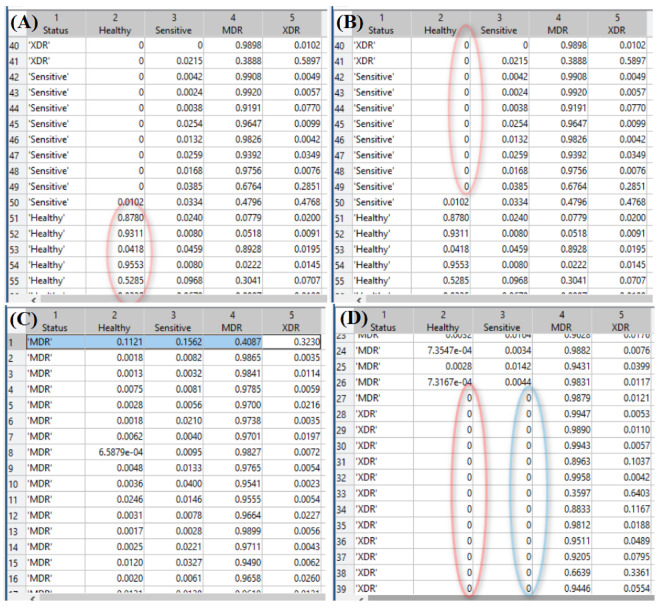
Examples from the table reporting probabilities assigned by the CatBoost multiclassifier with special highlighting of the maximal probabilities of the healthy status for the sample of healthy persons (**A**), zero probability of the “Healthy” status for persons suffering from MtB sensitivity to conventional drugs (**B**), no zero probability for multidrug-resistant case (**C**), and zero probability for persons suffering from extra drug-resistant MtB (**D**).

However, it was detected that the direct default application of the CatBoost multiclassifier evidently does not meet the reasonable acceptance criteria (see Figure 2A). Although it clearly distinguishes between healthy and infected people, the sensitivity to conventional drugs and the drug resistance type are not distinguished. Instead, the “naïve” application of the default method mainly classifies all biomarkers’ responses as belonging to the multidrug resistance case. This fact originates from the high range of scattering of the biomarker concentrations (see above) from which the multiclassifier denotes the intermediate status, as the most probable as can be seen by looking at the content of the CatBoost-generated file Out.tsv. Snapshots of these contents, formatted for better clarity, are shown in Figure 3, in which one can see the correct prevailing probability of the “Healthy” status for the inputs of the biomarker concentrations obtained from healthy persons, while, for all other cases, the largest probability is erroneously assigned to the status “MDR”.

Thus, there is a need for a deeper analysis of the multiclassification procedure and its improvement.

### 3.2. Improved Multiclassification

To correct the multiclassification procedure, let us consider probabilities, which CatBoost (“naive” approach) assigns to different classes as numbers explicitly.

Figure 3A demonstrates a part of the output of CatBoost in the form of a table in which the column “Status” is the actual status, and the four other columns report the probabilities assigned by CatBoost to the statuses listed in the columns’ headers. Here, one can see that the probability of the “Healthy” status is assigned in the majority of records. Thus, one can trust this output, as is seen in Figure 2A, in which all of the predicted markers of this class, except one, overlap with the actual one.

However, the situation with the prediction quality is drastically worse in the case of differentiation among various MtB drug resistance strains when CatBoost assigns MDR status to practically all data. Thus, the assigned probability should not be used directly in this case and requires special exploration. Figure 3B, illustrating the case of drug-sensible MtB, shows the completely erroneous maximal probability in the column ”MDR”. At the same time, one can see the specificity in the column ”Healthy”, which is filled by strict zeros. On the contrary, in the case of actual MDR in Figure 3C, all columns are filled with non-zero numbers. a certain regularity returns in the case of XDR MtB; one can see again that the columns ”Healthy” and ”Sensitive” are filled with the strict zeros in Figure 3D.

**Figure 4:**
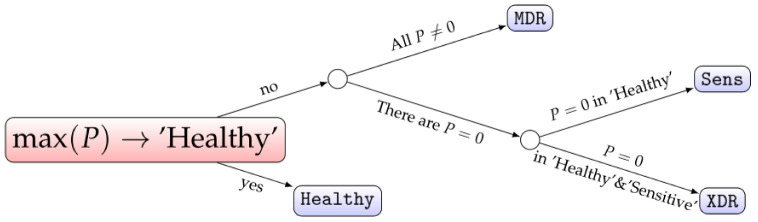
The decision tree summarising the workflow of determining the drug resistance status based on the postprocessing of raw values of probabilities assigned by the CatBoost multiclassifier.

Thus, we can state the existence of certain qualitative regularities in the values of probabilities assigned by the multiclassifier, which can serve as a better indicator of the drug resistance status than the quantitative values of these probabilities themselves. The proposed procedure is graphically summarised in the binary decision tree shown in Figure 4.

Let us discuss one more time the principal steps in coordination with the features of the biomarker concentration ranges shown in Figure 1. The root node corresponds to the default values of CatBoost’s probabilities reported by the multiclassifier function. However, the decision step for this node replaces the multiclassification with a binary classification that considers the choice between the healthy persons (when CatBoost assigns the maximal probability to this status) and infected persons (otherwise). The trustworthiness of this choice is biochemically based mainly on the values of the MMP-8 and MMP-9 concentrations (see Figure 1B,C, in which the respective plots demonstrate concentrations around zero). The alternative case requires consideration of the choice between the presence and absence of the strict zeros in CatBoost’s outcome probabilities. If there are no such zeros, the most probable actual status is the multidrug-resistant MtB. This high uncertainty in the outcome probabilities follows from the high range of the scattering of points in Figure 1. For example, Figure 1A shows that the cluster of MMP-1 points practically overlaps for healthy persons and patients with the sensitive and MDR forms of MtB. Figure 1B indicates densely placed points distributed with approximately the same concentration range for all three forms of MtB, and similar behaviour is expressed, though less pronouncedly, in Figure 1C.

Distinguishing between the two rest statuses is based on the binary choice between the number of strict zeros in CatBoost’s outcomes. When one such zero is assigned to the status ”Healthy”, one can conclude that the actual status is ”Sensitive” independently of the probability assigned by CatBoost. If the strict zero probabilities are assigned to both “uncomplicated” statuses, ”Healthy” and ”Sensitive”, the most probable actual status is the extra drug-resistant MtB even when CatBoost assigned the status MDR by default. This is biochemically explainable by exploring all sub-panels in Figure 1, where one can see a trend in the elevating concentrations of the biomarkers with growing drug resistance. Additionally, when the case of the ”Healthy” status is excluded, e.g., in contrast to the cases shown in Figure 1B,C, the prevalence of this trend determines the choice between ”XDR” and ”Sensitive”. In the former case, one expects that the concentration points shifted to the right-hand side of the axes of concentrations. If some overlapping with the concentrations of ”XDR” and ”Sensitive” occurs as, for example, in Figure 1D, then one excludes ”XDR” and assigns the ”Sensitive” status.

The results of applying the proposed decision tree-based procedure to the explored dataset are shown in Figure 2B. One can see a drastic improvement with respect to the default (naïve) case of multiclassification shown in Figure 2A. Now, 54 of 58 cases (i.e., 93%) have been predicted correctly.

Table 1 represents elements of the confusion matrix for separate classes of drug resistance: the true positive rate (TPR), also known as sensitivity or recall; the true negative rate (TNR), also known as specificity or selectivity; and the positive predictive value (PPV), also known as precision. Note that 100% of the results for TNR and PPV for healthy subjects originate from the absence of false positive results for this case (see Figure 2B).

One case of misprediction is found in each class; three of these warn about a more severe case than the real one and may be associated with the deviations in biomarker concentrations due to general health status.

Since the clinical data provided in [16] consists of a larger set that includes missing data for several values of concentrations of particular biomarkers, we carried out some additional tests to analyse the influence of missing (’NaN’) values in the input data. Note that CatBoost natively allows for processing of such datasets (both options –nan-mode Max and –nan-mode Max (default) were used, and there is no difference in their applications). In this case, we used 58 representatives without missing data as the training dataset and the data in which one of the parameters is missing as the test dataset (accounting for more missing data from four parameters is too sparse). The dataset, which may contain missing values in all fields except MMP-8, consists of 37 representatives (Healthy: 1, Sens: 3, MDR: 22, XDR: 11). The application of the proposed algorithm resulted in 51% accuracy of prediction. For the test sample of 98 representatives (Healthy: 2, Sens: 13, MDR: 55, XDR: 26), which may have a missing value anywhere except for MMP-9, the prediction accuracy is reduced to 41%. It is even further reduced to 39% and 33% when a missing value is located anywhere except for TIMP (64 representatives; Healthy: 2, Sens: 14, MDR: 55, XDR: 26) and MMP-1 (64 representatives; Healthy: 1, Sens: 12, MDR: 36, XDR: 15), respectively.

Thus, we can conclude that, although it is possible to indicate the different influences of input metabolic biomarkers on prediction accuracy, one should avoid using incomplete data when patients are blood-tested to obtain a highly accurate diagnosis.

## 4. Discussion

The method of decision trees enhanced with gradient boosting, one of the realisations of which is the considered approach, is one of the most in-demand machine learning approaches to diagnostics and treatment support of infectious diseases, including the drug resistance problem of TB [28,29,30,31]. The attractiveness of such an approach is based on the more interpretable character of the predictions, which are based on the subdivision of the input parameters’ ranges, in comparison with “black box” systems such as neural networks. At the same time, small samples with a high scattering of values can lead to a low accuracy when the default multiclassification is applied, as is demonstrated in the present work (the “naïve” approach). However, the general methodology is still valid, and the required accuracy can be elevated by the subsequent analysis of the qualitative character of the predictor’s values.

From a practical point of view, we can highlight not only the accuracy of predictions provided by the proposed methods but also the general usability of CatBoost as a computational system. In contrast to the alternative existing realizations, such as XGBoost, which normally require Python or R programming for the realisation of the computational procedure [32], there is native support of a command-line version of CatBoost that is quite simple to use. In fact, one needs only to form a text-based table with clinical data and run one line of command. Moreover, the output is also a text file, and thus, operating with the proposed improved method of multiclassification, it is possible to follow the tree-like choices based on the qualitative values of the output probabilities according to Figure 4, even in “hand mode”. In our opinion, this makes such an approach suitable for common clinicians, who need not master programming to be able to make conclusions about drug resistance by analysing the collected samples.

Finally, it is worth noting that the level of matrix metalloproteinases not only serves as a biomarker of the drug resistance of the *Mycobacterium tuberculosis* causing the illness but is directly related to its development and outcome. This induced a number of machine learning-based studies focused specifically on tissue destruction and treatment prognosis (see, e.g., [33,34]).

## 5. Conclusions

The main message of this work can be summarised as follows: the case of highly overlapping data, especially for the small samples typical of clinical data collections, can lead to strong misclassification when default multiclassification algorithms are applied. However, if considering the postprocessing of the outcome probabilities reported by the respective machine learning procedure, it is possible to improve prediction accuracy via the qualitative classification of these probabilities’ ranks.

By default, an output class is determined as that which corresponds to the maximal probability among all possible classes. At the same time, exploring the full table generated by the ML program allows more relevant qualitative patterns to be revealed. These patterns can be treated within a decision tree procedure that will result in an improvement in the predictive capacity.

In this work, this approach is evaluated via a case study of determining the drug resistance status of *M. tuberculosis* by processing the data on four biochemical markers (three types of matrix proteinases and the related tissue inhibitor TIMP) obtained from the blood tests of TB-suffering patients and a control group of healthy persons. We demonstrated that the proposed *CatBoost*-based algorithm provides an opportunity for improvement in the predictive capacity from the binary classification distinguishing between healthy and TB-suffering persons to the real multiclassification between different drug resistance statuses. Thus, the result is a step toward the fast diagnostics highly demanded by clinical practices when dealing with such a socially dangerous disease as tuberculosis.

At the same time, consideration of the ML-based results not as outputs of a “black box” but as a source for further exploration is not limited by this particular case and may be applied to more interpretable decisions in general.

## Figures and Tables

**Table 1:** Elements of the confusion matrix for the improved prediction procedure with to different drug resistance statuses.

Status	TPR, %	TNR, %	PPV, %
Healthy	87	100	100
Sens	89	98	89
MDR	96	93	93
XDR	93	98	93

## Data Availability

All biomedical data used in this work are freely available as Supplementary Material to the work [16].
